# Hydrogen bonding: a double-edged sword in aqueous ammonium-ion batteries

**DOI:** 10.1093/nsr/nwag490

**Published:** 2026-08-14

**Authors:** Runtian Zheng, Jie Shu, Yu Li, Bao-Lian Su

**Affiliations:** Laboratory of Inorganic Materials Chemistry (CMI), University of Namur, Belgium; School of Chemistry and Chemical Engineering, Shaoxing University, China; State Key Laboratory of Advanced Technology for Materials Synthesis and Processing, Wuhan University of Technology, China; Laboratory of Inorganic Materials Chemistry (CMI), University of Namur, Belgium; State Key Laboratory of Advanced Technology for Materials Synthesis and Processing, Wuhan University of Technology, China

## Abstract

Hydrogen bonding plays a double-edged role in ammonium-ion batteries, facilitating ion transport and flexible storage while inducing instability. Rational regulation of these interactions is crucial for achieving high-performance.

Hydrogen bonding represents one of the most fundamental and highly dynamic intermolecular interactions governing aqueous electrochemical systems. As a general X–H … A structure (where X is an electronegative donor and A is a lone-pair acceptor), hydrogen bonding arises primarily from the electrostatic attraction between the polarized H atom and the acceptor atom. As illustrated in Fig. 1, hydrogen bonding in aqueous ammonium-ion batteries (AIBs) extends beyond conventional solvent interactions, forming a dynamic multi-level network that connects water molecules, electrolyte anions, NH_4_^+^, and the electrode frameworks [1,2].

**Figure 1.**
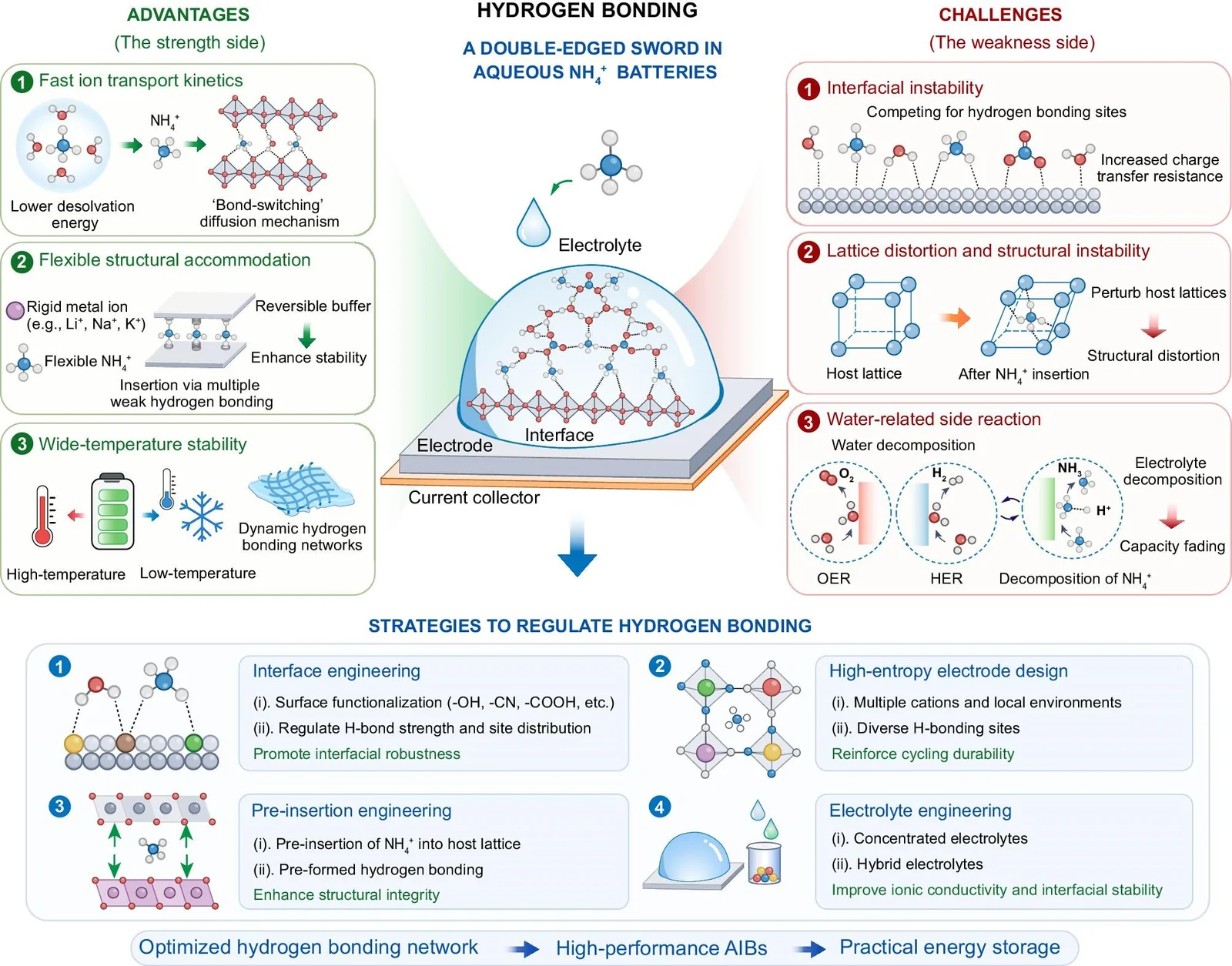
Advantages and challenges of hydrogen bonding in AIBs and strategies for regulation.

In contrast to the conventional coordination behavior of metal cations, NH_4_^+^ inserts into host frameworks through dynamic hydrogen bonding interactions rather than rigid ionic coordination [3–5]. As a result, the electrochemical behavior is governed by reversible hydrogen bonding interactions involving both electrolyte species and electrode frameworks. Given its positive charge, NH_4_^+^ exhibits strong electrostatic affinity toward electron-rich O/N sites, positioning it as both a charge carrier and a hydrogen bonding donor within the system.

Although hydrogen bonding underpins the exceptional kinetic advantages of NH_4_^+^ storage systems, its dynamic and evolving nature can also lead to interfacial instability, lattice distortion, structural degradation, and side reactions during prolonged cycling. Therefore, hydrogen bonding should be viewed as a double-edged sword, simultaneously governing both the superior electrochemical kinetics and the intrinsic stability limitation.

The dynamic hydrogen bonding network endows NH_4_^+^ storage systems with distinct advantages, enabling rapid ion transport, structural flexibility, and stable operation across a broad temperature range. These unique characteristics are discussed in the following sections.

**Fast ion transport kinetics.** In aqueous electrolytes, NH_4_^+^ exhibits fast transport kinetics owing to its hydrogen-bonding-dominated solvation structure. Unlike strongly coordinated metal ions such as Li^+^, Na^+^, and K^+^, NH_4_^+^ interacts with H_2_O through directional N–H … O hydrogen bonding, forming a compact and highly dynamic solvation shell [6]. This results in a smaller effective hydrated radius and reduced desolvation energy, enabling fast ion migration across the electrolyte–electrode interface.

For electrode frameworks, the diffusion of NH_4_^+^ is further facilitated by dynamic hydrogen bonding rearrangements. During insertion, its N–H bonds continuously deform under local electrostatic fields, forming and breaking transient hydrogen bonding interactions with lattice O/N/S sites. This ‘bond-switching’ diffusion mechanism, rather than rigid site-to-site hopping, significantly lowers migration barriers and enhances rate performance [7].

**Flexible structural accommodation.** Owing to its flexible tetrahedral geometry, NH_4_^+^ possesses N–H bonds capable of dynamically adapting to the local structural environment during transport and insertion. Unlike rigid metal cations, NH_4_^+^ interacts with host frameworks through multiple weak hydrogen bonding interactions. In materials such as metal oxides, NH_4_^+^ insertion proceeds via reversible hydrogen bonding rearrangement rather than strong ionic coordination. Consequently, hydrogen bonding acts as a reversible buffer that enhances cycling stability and structural robustness.

**Wide-temperature stability.** Hydrogen bonding also enables NH_4_^+^-based systems to operate over a broad temperature range. Since NH_4_^+^ transport is governed by dynamic hydrogen bonding networks, the solvation structure exhibits adaptability under thermal variation. Especially at low temperatures, hydrogen bonding prevents complete freezing of the solvation sheath, thus maintaining partial ion mobility. Consequently, NH_4_^+^ transport has weak temperature dependence, ensuring stable electrochemical performance under extreme conditions.

Despite these advantages, hydrogen bonding interactions that enhance kinetics and structural adaptability can also induce instability. The following section discusses their impact on electrochemical performance and stability.

**Interfacial instability.** The hydrogen-bonding-rich environment also introduces significant interfacial challenges. Strong coupling between NH_4_^+^ and H_2_O could alter local proton activity, promoting side reactions, particularly at high potentials. Moreover, the competition among NH_4_^+^, H_2_O, and anions for hydrogen bonding sites may induce interfacial disorder, leading to ill-defined adsorption configurations and an increased charge-transfer resistance. Such interfacial disorder could hinder charge-transfer kinetics and block active redox sites, thereby reducing the accessible capacity and Coulombic efficiency.

**Lattice distortion and structural instability.** Although hydrogen bonding facilitates NH_4_^+^ accommodation, it could also disrupt host lattices. NH_4_^+^ forms extensive hydrogen bonding interactions with lattice O/N atoms, altering local bond lengths and angles during its insertion and extraction processes [8]. Such continuous perturbation accumulates as microstrain, leading to subtle structural distortion and partial framework decomposition. This behavior contrasts with the more rigid coordination observed in metal-ion systems, highlighting the unique yet complex nature of its storage chemistry.

**Water-related side reactions.** In aqueous electrolytes, NH_4_^+^ is strongly hydrated, and the resulting hydrogen bonding network serves as a major pathway for H_2_O decomposition. This promotes the oxygen evolution reaction (OER) and hydrogen evolution reaction (HER), thereby narrowing the electrochemical stability window. Furthermore, NH_4_^+^ may undergo partial hydrolysis to generate NH_3_ and H^+^, which lowers the local pH and further accelerates HER. These processes reduce Coulombic efficiency, degrade cycling stability, and limit the compatibility of aqueous electrolytes with high-voltage electrodes.

A balance between the beneficial and adverse roles of hydrogen bonding can be achieved through targeted regulation of the battery system. Accordingly, a variety of strategies have been developed to manipulate hydrogen bonding interactions across different components, including electrode interfaces, host frameworks, and electrolyte compositions.

**Interface engineering.** Surface functional groups such as −OH, −CN, and −COOH can further modulate NH_4_^+^ adsorption strength through controlled hydrogen bonding. By tailoring the hydrogen bonding strength and site distribution at the interface, interfacial resistance is reduced, thereby enhancing kinetic performance. The key design principle, therefore, is not to eliminate hydrogen bonding but to confine it within an optimal energetic window—one that ensures fast kinetics while avoiding interfacial blockage.

**High-entropy electrode design.** The high-entropy strategy leverages multiple cations to engineer heterogeneous local environments with diversified hydrogen bonding sites. Moreover, this design promotes more balanced NH_4_^+^ host interactions, alleviates local stress accumulation arising from repeated hydrogen bonding formation and dissociation, and stabilizes the crystal framework against structural degradation [9]. Collectively, these effects translate into enhanced structural stability and prolonged cycling durability.

**Pre-insertion engineering.** Pre-insertion of NH_4_^+^ into host frameworks establishes a pre-formed hydrogen bonding network that stabilizes the lattice. By buffering volumetric strain, enabling more reversible ion diffusion, and mitigating microstrain accumulation during cycling, this pre-conditioned structure enhances long-term structural stability.

**Electrolyte engineering.** Electrolyte composition and concentration strongly influence the hydrogen bonding structure. Salts such as NH_4_(CH_3_COO), NH_4_Cl, and (NH_4_)_2_SO_4_ modify local polarity and thus regulate interactions among NH_4_^+^, water, and electrode surfaces [10]. In particular, highly concentrated ‘water-in-salt’ electrolytes markedly reduce water activity and tailor the solvation structure, while hybrid and mixed-anion electrolytes optimize interfacial hydrogen bonding interactions, thereby enhancing ionic conductivity and interfacial stability. Furthermore, advances in solid-state electrolytes for proton batteries offer important guidance for the rational design and long-term stabilization of electrolytes for NH_4_^+^ storage [11,12].

Hydrogen bonding plays a central role in NH_4_^+^ transport, interfacial chemistry, and structural evolution in AIBs. The key challenge is to balance the dual nature of hydrogen bonding, preserving its dynamic reversibility while mitigating disorder and instability. To address this challenge, future efforts should combine advanced multiscale characterization, simulations, theoretical calculations, and artificial-intelligence-driven predictive tools to resolve hydrogen bonding dynamics during electrochemical processes. Such mechanistic insights will enable rational network design, turning limiting factors into tunable parameters for next-generation AIBs. More broadly, regulating hydrogen bonding offers a versatile strategy for controlling ion-host interactions, providing valuable guidance for the rational design of advanced electrode materials across diverse aqueous ion-storage systems.

## References

[1] Kulkarni A, Padwal C, MacLeod J et al. Adv Energy Mater 2024; 14: 2400702. 10.1002/aenm.202400702

[2] Zheng R, Ding Y, Zhang Y et al. Angew Chem Int Ed 2026; 65: e6628239. 10.1002/anie.6628239 PMC13452555 42261912

[3] Zhao C, Yan J, Ma Z et al. Chem Synth 2025; 5: 32. 10.20517/cs.2024.14

[4] Chen YX, Chen R, Lu CZ. Chem Synth 2024; 4: 74. 10.20517/cs.2024.109

[5] Lin YT, Lin BL, Niu BT et al. Chem Synth 2025; 5: 37. 10.20517/cs.2024.53

[6] Wessells CD, Peddada SV, McDowell MT et al. J Electrochem Soc 2011; 159: A98–103. 10.1149/2.060202jes

[7] Dong S, Shin W, Jiang H et al. Chem 2019; 5: 1537–51. 10.1016/j.chempr.2019.03.009

[8] Zhou M, Huang X, Li H et al. Angew Chem Int Ed 2024; 63: e202413354. 10.1002/anie.202413354 39157909

[9] Zou J, Wang C, Xu B et al. Small 2025; 21: 2501370. 10.1002/smll.202501370 40103453

[10] Bao Z, Lu C, Liu Q et al. Nat Commun 2024; 15: 1934. 10.1038/s41467-024-46317-5 PMC10908845 38431736

[11] Qiao Q, Ni XQ, Xiong X et al. Angew Chem Int Ed 2026; 65: e19623. 10.1002/anie.202519623 41217091

[12] Anwar MF, Yu Y, Khalid M et al. Energy Mater 2025; 5: 500102. 10.20517/energymater.2025.16

